# Pupil diameter reflects uncertainty in attentional selection during visual search

**DOI:** 10.3389/fnhum.2015.00435

**Published:** 2015-08-04

**Authors:** Joy J. Geng, Zachary Blumenfeld, Terence L. Tyson, Michael J. Minzenberg

**Affiliations:** ^1^Center for Mind and Brain and Department of Psychology, University of California, DavisDavis, CA, USA; ^2^Department of Psychology, University of California DavisDavis, CA, USA; ^3^Department of Psychiatry, University of California San Francisco School of MedicineSan Francisco, CA, USA

**Keywords:** pupillometry, visual search, cognitive control, attentional selection, LC-NE system, uncertainty

## Abstract

Pupil diameter has long been used as a metric of cognitive processing. However, recent advances suggest that the cognitive sources of change in pupil size may reflect LC-NE function and the calculation of unexpected uncertainty in decision processes (Aston-Jones and Cohen, 2005; Yu and Dayan, 2005). In the current experiments, we explored the role of uncertainty in attentional selection on task-evoked changes in pupil diameter during visual search. We found that task-evoked changes in pupil diameter were related to uncertainty during attentional selection as measured by reaction time (RT) and performance accuracy (Experiments 1-2). Control analyses demonstrated that the results are unlikely to be due to error monitoring or response uncertainty. Our results suggest that pupil diameter can be used as an implicit metric of uncertainty in ongoing attentional selection requiring effortful control processes.

## Introduction

Visual search tasks are commonly used to understand the mechanisms of selective attention because of their clear ecological validity. For example, the daily act of going to work involves a sequence of visual search tasks in which you must first find the right clothes, breakfast items, then keys, etc. During this process, you will inevitably attend to a series of non-target objects (particularly ones that are salient or target-similar) before the final objects of interest are found (Treisman and Gelade, 1980; Duncan and Humphreys, 1989; Wolfe and Horowitz, 2004). However, at each moment, there may be different degrees of uncertainty associated with the selection of each object. This uncertainty may arise from internal sources, such as a lapse of attention, or may come from external sources, such as a distraction or change in lighting that increases perceptual difficulty. In all cases, the engagement of cognitive control mechanisms must be increased to reinstate goal representations and determine the object's task relevance.

In the reported experiments, we used changes in pupil diameter as a metric of ongoing uncertainty during visual search. The use of pupils to index ongoing cognitive processes has a long history. For example, Kahneman and Beatty (1966) reported larger changes in pupil size with increased “mental effort,” defined by the quantity of information held in working memory or the complexity of mental manipulations made on information in memory (Kahneman and Beatty, 1966; Beatty, 1982). Similarly, Richer and Beatty (1987) found pupil size to be related to “response uncertainty” in a task for which subjects were asked to map four manual responses to four different tones compared to just one or two responses to the same four tones. Pupil size was larger in the four-response condition even when the execution of a manual response and its reaction time (RT) were accounted for, suggesting that the changes in pupil were based on the cognitive demands of selecting the correct response rather than the response itself (Richer and Beatty, 1987; Einhäuser et al., 2010; Privitera et al., 2010; Wierda et al., 2012). These studies established the idea that task-evoked changes in pupil size reflect the ongoing cognitive demands of a task, including the number of items held in memory, the difficulty of the task, or the number of possible alternative responses.

More recently, these findings have been extended to demonstrate that task-evoked changes in pupil size correlate with a more varied set of cognitive processes, including conflict processing, surprise, target detection, working memory, attention, and awareness (Einhäuser et al., 2008; Chatham et al., 2009; Privitera et al., 2010; Gabay et al., 2011; Preuschoff et al., 2011; Laeng et al., 2012; Wierda et al., 2012; Chiew and Braver, 2013; Payzan-LeNestour et al., 2013; Binda et al., 2014; Johnson et al., 2014; Lavín et al., 2014; Unsworth and Robison, 2015). For example Johnson et al. (2014) found that in both children and adults, pupil size increased during a digit span task until short-term memory was overloaded, at which point subjects disengaged and their pupil diameter began to decrease. This result suggested that pupil diameter could be used as a continuous metric of ongoing cognitive processes within a single trial and to identify the point at which cognitive control processes are disengaged.

Similarly, Geva et al. (2013) demonstrated that even within a trial, different components of attentional control processes could be decoded from the task-evoked pupil diameter. Using the Attention Network Task (ANT) (Posner and Petersen, 1990; Petersen and Posner, 2012), Geva et al. (2013) found an early attentional orienting component 360 ms following an attentional cue and a later executive control component occurring around 1200 ms following the target. The amplitude of the later component was larger on conflict trials when the target appeared with incongruent flankers compared to congruent or neutral flankers, suggesting sensitivity to conflict processing; furthermore, pupil diameter on incongruent trials was estimated to be three times larger when an error was made compared to a correct response, suggesting that larger pupil diameter on incongruent and error trials reflected similar mechanisms of effortful performance monitoring that were most extreme when errors were actually made.

Although the cognitive processes that produce reliable changes in pupil diameter appear varied, they have been united by a mechanistic neuromodulatory model showing that changes in pupil diameter covary with locus coeruleus (LC)-norepinephrine (NE) system activity (Aston-jones et al., 1994; Usher et al., 1999; Aston-Jones and Cohen, 2005; Cohen and Aston-Jones, 2005; Yu and Dayan, 2005; Bari and Aston-Jones, 2013). Aston-Jones and colleagues found that changes in the mode of neuronal firing in the LC, the sole nucleus responsible for NE release throughout the brain, was correlated with changes in both baseline and task-evoked pupil size; furthermore, these changes covaried with shifts in behavioral strategies that favored task exploitation (i.e., using known statistics to maximize performance efficacy) vs. exploration (i.e., learning or updating expectations about the environment) (Gilzenrat et al., 2010; Bari and Aston-Jones, 2013; Eldar et al., 2013). Their theory of adaptive gain proposes that LC-NE activity optimizes behavior by adjusting strategic cognitive processing mediated by LC projections to prefrontal cortex, and that pupil diameter reflects these strategic changes (Beracochea et al., 2001; de Saint Hilaire et al., 2001; Piérard et al., 2006; Minzenberg et al., 2008; Ambrosini et al., 2013; Marzo et al., 2014).

A particularly interesting consequence of this work by Aston-Jones, Cohen, and colleagues has been the development of more precise ways to understand how NE modulates cognition (Yu and Dayan, 2005; Rushworth and Behrens, 2008; Yu et al., 2009; Gilzenrat et al., 2010; Chiew and Braver, 2013; Eldar et al., 2013; Lavín et al., 2014). One particularly influential idea has been that NE may signal the presence of unexpected uncertainty in the environment (Yu and Dayan, 2005). Unexpected uncertainty, which can be understood as information that violates expectations about the environment or stimuli, can be contrasted with expected uncertainty, which reflects known volatility in the environment (Behrens et al., 2007; Preuschoff et al., 2011). Thus, unexpected uncertainty might broadly be considered as a signal that indicates instability in current models of the world and a need to acquire more information. This general framework fits well within visual search paradigms from the attentional literature in which there is uncertainty associated with the selection of subsequent items during a temporally elongated search process for a particular target object.

In the current studies, we hypothesized that changes in pupil size would reflect the degree of uncertainty during visual search for a target. We expected uncertainty to be triggered by changes in both the task structure and variability in internal mental processes such as mind-wandering or other lapses in attentional focus (Weissman et al., 2006a; Smallwood et al., 2011; Smallwood and Schooler, 2015). Surprisingly, we did not find reliable changes in pupil size related to the task conditions, but did find large effects related to variability in performance and task difficulty. We conclude that the evoked pupil diameter reflects uncertainty during attentional selection that is triggered primarily by internal fluctuations in attentiveness; and that the uncertainty initiates the involvement of prefrontal cognitive control mechanisms to help disambiguate sensory information and determine the correct response.

## Experiment 1AB

**Figure 1:**
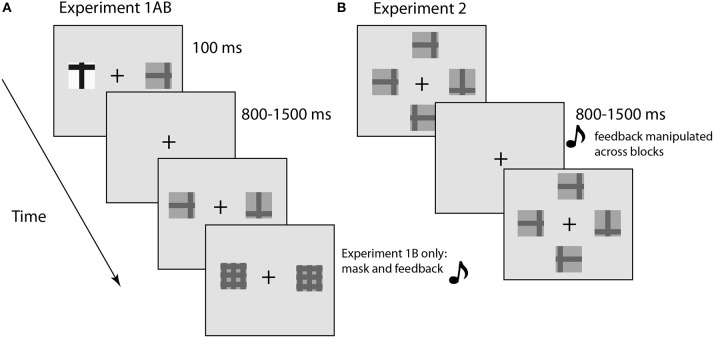
**Illustration of experimental design**. **(A)** E1A and E1B involved visual search for a target in the presence of a single distractor. The target was designated the “t” and subjects discriminated the orientation (upright or inverted). E1B was identical to E1A with the addition of a pattern mask and auditory feedback. **(B)** E2 was similar to E1AB, but included two additional distractors to increase task difficulty. The duration of the display was determined for each subject using the method of constant stimuli. Feedback was manipulated across blocks.

Subjects were asked to engage in a visual search task in which the probability of targets or distractors appearing in a perceptually salient feature (high contrast) was manipulated over blocks (Figure 1). We hypothesized that there would be changes in the task-evoked pupil diameter when observers experienced greater uncertainty in the search process, due either to changes in the task structure (indicating a need to learn new stimulus statistics) or in response to lapses of attention that would increase RT and the likelihood of a behavioral error (Weissman et al., 2006a; Geng and Mangun, 2009; Smallwood et al., 2011). Experiment 1B differed from 1A in two ways: first, we used a post-stimulus pattern mask to better control the duration of sensory processing; and second, we introduced auditory feedback after the manual response to reduce uncertainty about the accuracy of the response itself. Experiment 1B was a conceptual replication of 1A and therefore the results are presented together.

### Methods

#### Participants

Sixteen individuals (ten female, three left-handed overall) participated in Experiment 1A and 15 different individuals participated in Experiment 1B (seven female, all right-handed). All were from the University of California, Davis. Subjects were between 18 and 25 years old. Each received course credit for participating. Four participants from E1A and two from E1B were excluded based on poor performance (i.e., overall accuracy under 70%), leaving 12 subjects in the final analysis of Experiment 1A and 13 in 1B. The experimental protocol was approved by the Institutional Review Board at UC Davis.

#### Apparatus and stimuli

Visual search displays contained one or two “t” stimuli. The target was an upright or inverted “t” and the distractor, when present, was a 90° rotated version of the same stimulus (Figure 1). The stimuli were either “neutral” (i.e., a low contrast stimulus; Michelson Contrast Ratio = 0.51; foreground luminance = 5.4 cd/m^2^, background luminance = 16.8 cd/m^2^), or salient (i.e., a high contrast stimulus; Michelson Contrast Ratio = 0.96; foreground luminance = 0.54 cd/m^2^, background luminance = 30.5 cd/m^2^) (Figure 1). The background was a neutral gray color (9.8 cd/m^2^), with a black fixation cross in the center of the screen. Subjects pressed the “j” key when the target was upright and the “n” key when it was inverted. Subjects were instructed to be as fast and accurate as possible. The stimulus objects were presented on the horizontal meridian of the screen at 5.2° visual angle (near edge) and 6.57° visual angle (outer edge) from fixation. Pupil diameter was recorded with an Eyelink 1000 (SR Research) sampling at 500 Hz.

In addition, in Experiment 1B, a pattern mask followed the visual search display and an auditory tone provided immediate performance feedback. The tone lasted for 200 ms (correct = 800 Hz; error = 200 Hz). The stimulus onset asynchrony (SOA) between the stimulus and the pattern mask was determined before the experiment for each subject using the method of constant stimuli. During this procedure, only neutral targets and distractors were displayed with the following SOAs: 70, 120, 170, 220, and 270 ms. The SOA with 75–80% accuracy at the end of the staircase was used throughout the main experiment. The average SOA was 105 ms (±17 ms based on the 60 Hz monitor refresh rate).

#### Procedure

On each trial, a target appeared either alone (17% of trials) or with a distractor. The target-alone trials were included to increase distractor uncertainty (i.e., operate as “catch trials”) that were not conditions of primary interest; these trials were therefore excluded from the analyses of interest. The target had an equal likelihood of appearing on either the left or the right side of a fixation cross and was either neutral or salient. The distractor always had the opposite saliency. The probability of the salient object being the distractor (compared to the target) was manipulated across four blocks: 0, 30, 70, and 100%. Block ordering was fixed so that the expectation of a salient distractor grew over the course of the experiment. The blocks with 0% and 100% salient distractors (i.e., blocks 1 and 4) contained 72 trials each; blocks with 30 and 70% salient distractors (i.e., blocks 2 and 3) each contained 108 trials in order to maximize the number of trials in the less frequent condition. Because of the probability manipulation across blocks, only blocks 2 and 3 included the two main trial types of interest: neutral target + salient distractor and salient target + neutral distractor trials. Previous work has demonstrated that salient distractors interfere with visual search if they are unexpected, but when expected can actually facilitate target search (Geng and DiQuattro, 2010). Therefore, we expected salient distractors to interfere with performance less in block 3, when they were more frequent, than in block 2, when they were less frequent.

Participants were seated approximately 600 mm in front of the display monitor in a dimly illuminated room. Subjects were asked to maintain fixation on the central cross throughout the experiment, and each trial began only after central fixation was detected by the eye-tracker for 100 ms. This procedure ensured that subjects were looking directly at the fixation cross when the search display appeared. The search display was visible for 100 ms (±17 ms based on the 60 Hz refresh rate of the monitor) and replaced by a blank screen with the fixation cross until a manual response was made. The same fixation screen remained on for an additional variable inter-trial-interval (ITI) of 800–1500 ms before the next trial began. This additional time was introduced to allow continued monitoring of the pupil beyond the response. Each subject performed 24 practice trials before the main experiment. There were 30 blocks of 12 trials each in the main experiment. Break periods occurred after the tenth and nineteenth trial blocks.

#### Analysis of pupil data

All pupil data were normalized to the grand mean over the entire experiment for each individual. Blinks were removed by the Eyelink parser blink detection algorithm, which identifies blinks as periods of loss in pupil data surrounded by saccade detection, presumed to occur based on the sweep of the eyelid during the closing and opening of the eye. Saccades were defined by a 30°/s velocity threshold. Additionally, in order to account for any differences in baseline pupil size between blocks or conditions, the stimulus-evoked data for each subject was normalized to the baseline period 1000 ms prior to stimulus onset.

After establishing the temporal characteristics of the pupil time series using principal components analysis (PCA), differences in pupil data between two conditions was analyzed using a non-parametric permutation approach that estimated the distribution of values expected from noise from the current dataset alone. This approach involves repeated permutation of the original data in order to estimate the probability that patterns in the data are due to the experimental manipulation rather than due to random variation in the data (Ernst, 2004; Sawaki et al., 2012). For each comparison of interest, the trial-averaged pupil time-series from one condition was subtracted from the other in order to generate a time-series of difference values. The data were then averaged per subject over the relevant time window in order to generate a single value of pupil diameter in that condition. For the permutation analysis, the data from each subject were randomly assigned to each of the conditions and treated in exactly the same manner as the real data. This was done 5000 times. An effect in the real data was considered significant if it had a <5% chance of occurring in the permuted data.

#### Temporal characteristics of pupil diameter

Previous work has shown that the pupil response contains sensory as well as cognitive components (Hoeks and Levelt, 1993; Wierda et al., 2012; Geva et al., 2013). In order to identify the sensory and cognitive components within the pupil data, we subjected the data from each experiment, time-locked to stimulus onset, to a PCA (Siegle et al., 2001; Kuchinke et al., 2007). PCA is an exploratory method that allowed us to identify the critical structure of the pupil time series. The average pupil dilation for each subject and in each condition was considered a variable; this produced a time × person and condition matrix. Factors represented groups of time points with high bivariate correlations and Varimax rotation was used to produce orthogonal factors.

**Figure 2:**
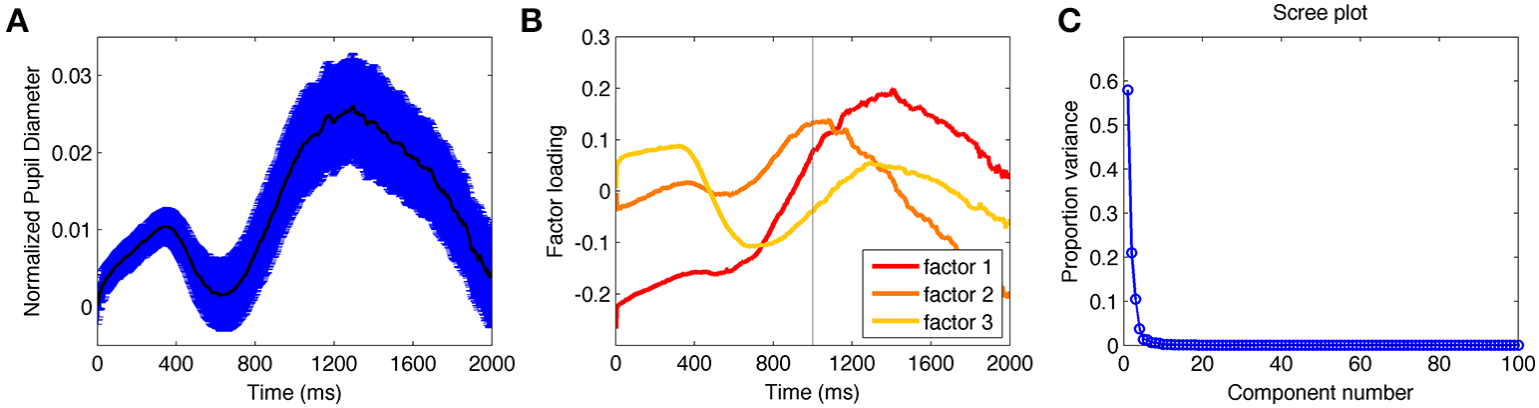
**(A)** Average pupil diameter from all conditions over time from E1AB. **(B)** Factor loadings for the first three extracted components from the principle component analysis. **(C)** Scree plot indicating the proportion of variance accounted for each factor (calculated from each eigenvalue divided by the sum of all eigenvalues).

The grand average pupil diameter time series visibly contained an early increase and decrease followed by a more sustained positive increase (Figure 2A). Statistical comparisons of the factor scores derived through PCA were used to identify the significance of these temporal characteristics. In both E1A and E1B, the first three factors were the only ones that independently explained more than 5% of the variance; together they accounted for 87% (E1A) and 92% (E1B) of the variance (Figure 2C).

PCA identified one factor characterizing the early dip (factor 3), and two factors characterizing the later increase in pupil size (factors 1 and 2) (Figure 2B). The early dip was consistent with the timing of sensory-evoked changes in pupil diameter (Hoeks and Levelt, 1993; Geva et al., 2013). Consistent with this, the factor loadings on this component showed a significant effect of condition, E1A: *F*_(3, 33)_ = 2.9, *p* < 0.05; E1B = *F*_(3, 36)_ = 41.5, *p* < 0.001, with significant differences between the neutral and salient target-only conditions, E1A: *t*_(11)_ = 2.3, *p* < 0.05; E1B: *t*_(12)_ = 4.6, *p* < 0.001; and no difference between the two two-stimuli conditions, which were equated in luminance, E1A: *t*_(11)_ = 1.5, *p* > 0.14; E1B: *t*_(12)_ = 0.15, *p* > 0.5. These data confirmed that the early component reflected sensory-evoked changes driven by the sensory properties of stimuli on the screen and not by the identity of the stimuli as targets or distractors.

There were no significant effects of condition on factors 1 and 2 in E1A. There was a significant effect of condition on factor loadings 1 and 2 in E1B, both *F*_(3, 36)_ = 4.6, *p* < 0.01. However, the factor loadings did not differ based on sensory properties as they did for factor 3. The significant differences were driven by the values in the two neutral-target conditions being larger than the salient-target conditions for factor 1, both *t*_(12)_ > 2.6, *p* < 0.05; and the two two-stimulus conditions being larger than the target-alone conditions in factor 2, both *t*_(12)_ < 2.2, *p* < 0.05. This suggests that the effects of condition on factors 1 and 2 in E1B were not sensory-based, but depended on cognitive factors associated with search.

Based on the PCA results, we divided the time series data into two time periods: an earlier “sensory” component from 1 to 1000 ms and a later “cognitive” component from 1001 to 2000 ms. Dividing the data into these two time periods is imperfect, but affords the advantage of analyzing pupil diameter in the cognitive period and comparing those results against sensory period as control data from within the same trial. Using fixed time periods also prevents potential differences in factor components derived from the data based on each comparison of interest from producing spurious inhomogeneity in interpreting the results. Furthermore, this approach is conservative and will be more likely to result in type II errors, given that the temporal division is imperfect.

### Results

The main goal of the experiment was to examine the relationship between changes in pupil size and uncertainty in attentional selection. We hypothesized that there would be two sources of uncertainty in attentional selection: (a) changes in the probability of the target (and distractor) being visually salient over blocks; and (b) internal fluctuations in attentiveness throughout the experiment that are reflected in RT and accuracy. To our surprise, there were no effects due to the manipulation of salience, but there were large and systematic pupil changes due to fluctuations in performance as measured by RT and accuracy. The results for the task-based effects are reported first followed by analyses of variability in performance.

#### Task-based effects of saliency across blocks

**Figure 3:**
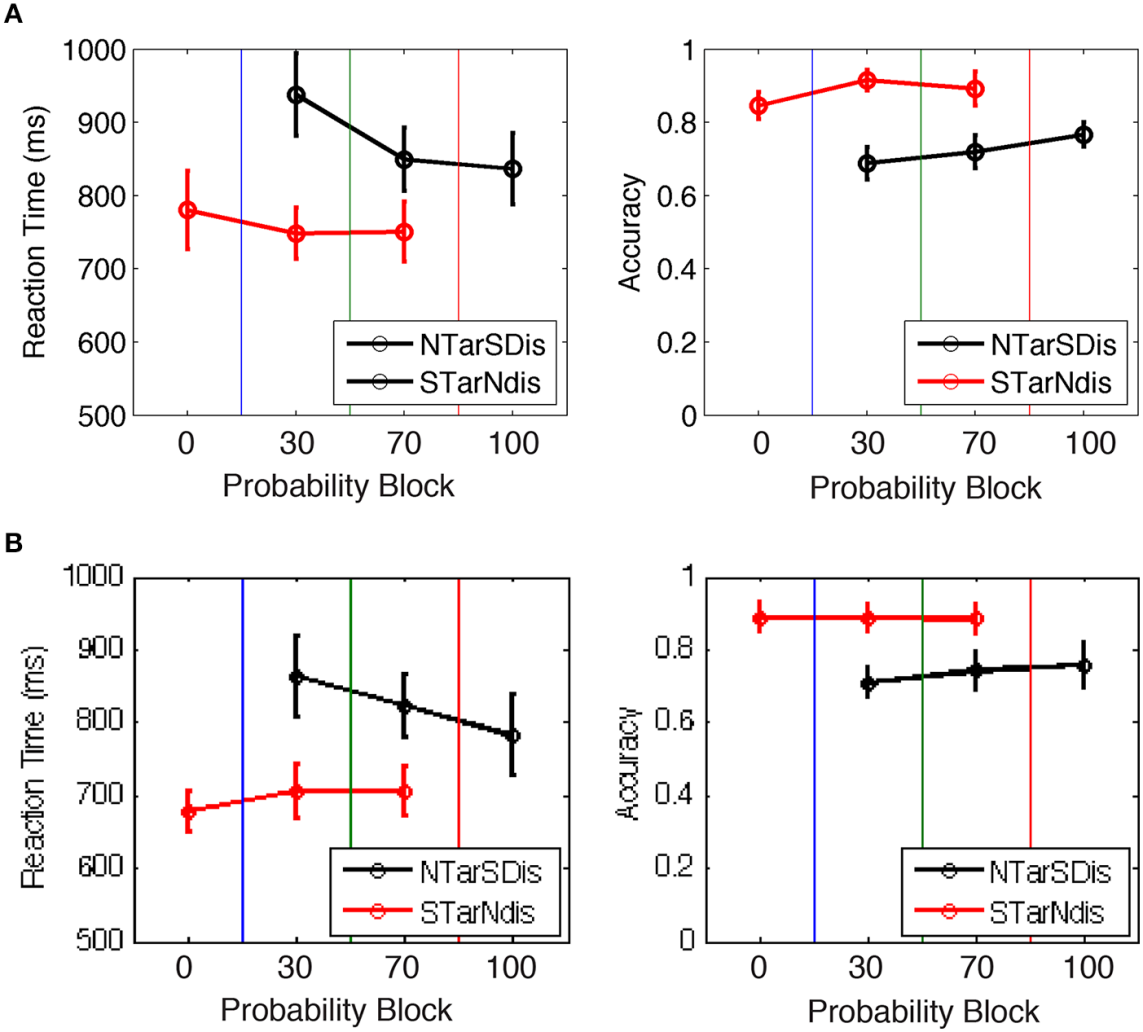
**Behavioral performance for Experiments (A) 1A and (B) 1B, showing that RT and accuracy changed with the manipulation of target vs. distractor saliency**. NTarSDis, neutral target + salient distractor; NTar, neutral target alone; STarNdis, salient target + neutral distractor; star, salient target alone.

We hypothesized that trials with salient distractors would produce greater uncertainty and longer RTs due to competition between top-down and bottom-up selection mechanisms, particularly in block 2 when salient distractors were novel and infrequent compared to block 3 when salient distractors were common. The behavioral data from blocks 2 and 3, which contained trials with both neutral and salient targets (and distractors with opposite saliency), were entered into a repeated measures ANOVA with block (2, 3) and target salience (neutral, salient) as fixed-effects factors. The main effect of salience and the interaction between salience and block were found in both experiments [E1A: salience: *F*_(1, 11)_ = 11.1, *p* < 0.01, block: *F*_(1, 11)_ = 12.5, *p* < 0.005, interaction: *F*_(1, 11)_ = 7.6, *p* < 0.05; E1B: salience: *F*_(1, 12)_ = 16.1, *p* < 0.005, block: *F*_(1, 12)_ = 1.2, *p* = 0.29, interaction: *F*_(1, 12)_ = 21.4, *p* < 0.001]. The significant interaction in both experiments was due to larger differences in RT between saliency conditions in block 2 compared to block 3 (Figure 3). Similar effects were obtained in the accuracy data [E1A: block *F*_(1, 11)_ = 0.03, *p* > 0.8, salience *F*_(1, 11)_ = 28.5, *p* < 0.001, interaction *F*_(1, 11)_ = 1.3, *p* > 0.3; E1B: block *F*_(1, 12)_ = 0.35, *p* > 0.5, salience *F*_(1, 12)_ = 11.3, *p* < 0.01, interaction *F*_(1, 12)_ = 5.0, *p* < 0.005]. The behavioral results were consistent with our expectations that the appearance of infrequent salient distractors would interfere more with performance when they were novel in block 2 compared to when they were more expected in block 3.

In order to evaluate the effect of uncertainty in response to seeing a salient distractor, we compared the evoked pupil diameter between the target- and distractor-salient conditions within each block as well as each condition across blocks. This resulted in four comparisons: two comparisons of the target- and distractor-salient conditions within block 2 and block 3 separately; and two comparisons of each saliency condition across blocks. These comparisons are equivalent to the relevant pairwise comparisons from the 2 × 2 ANOVA used for analyzing the associated behavioral data (see above). Using the permutation approach (described above), none of the differences were significant: all had a >20% probability of being due to chance. This result was unexpected and demonstrated that the novelty of the salient stimulus appearing for the first time in block 2 as a distractor did not evoke stimulus-driven changes in pupil diameter even though the manipulation affected behavioral performance.

The pre-stimulus pupil diameter has been used as a metric of the baseline activity in the LC-NE system. Therefore, we compared pupil diameter (1000 ms prior to stimulus onset) between blocks 1 and 2, as an additional test of pupil differences during the transition in salience association. Because trials were randomized with regard to condition, trials in block 2 that preceded evidence that distractors could be salient were excluded from the analysis. There was no significant difference in either experiment {E1A: *t*_(11)_ = 0.2, *p* = 0.84, mean difference [95% CI] = 0.003[−0.03, 0.04]; E1B: *t*_(12)_ = 1.4, *p* = 0.18, mean difference [95% CI] = −0.03[−0.08, 0.02]}. In order to test the possibility that any differences in the pre-stimulus pupil only were masked by the end of block 2, we also compared the pupil data from block 1 to the first 12 post-change trials in block 2; the results were also not significant {E1A: *t*_(11)_ = 1.34, *p* = 0.2, mean difference [95% CI] = −0.04[−0.1, 0.02]; E1B: *t*_(12)_ = 1.3, *p* = 0.21, mean difference [95% CI] = −0.03 [−0.08, 0.02]}. These data suggested that although salient distractors interfered with performance, they did not induce systematic changes in the baseline or the stimulus-evoked pupil diameter. It may be that the saliency manipulation did not increase selection uncertainty sufficiently to require systematic changes in cognitive control mechanisms indicated by pupil diameter, or simply that our measurements were insensitive to the size of changes that occurred. We next examined the effects of internal sources of variability in attentiveness that would also affect selection uncertainty.

#### Performance variability and accuracy

**Figure 4:**
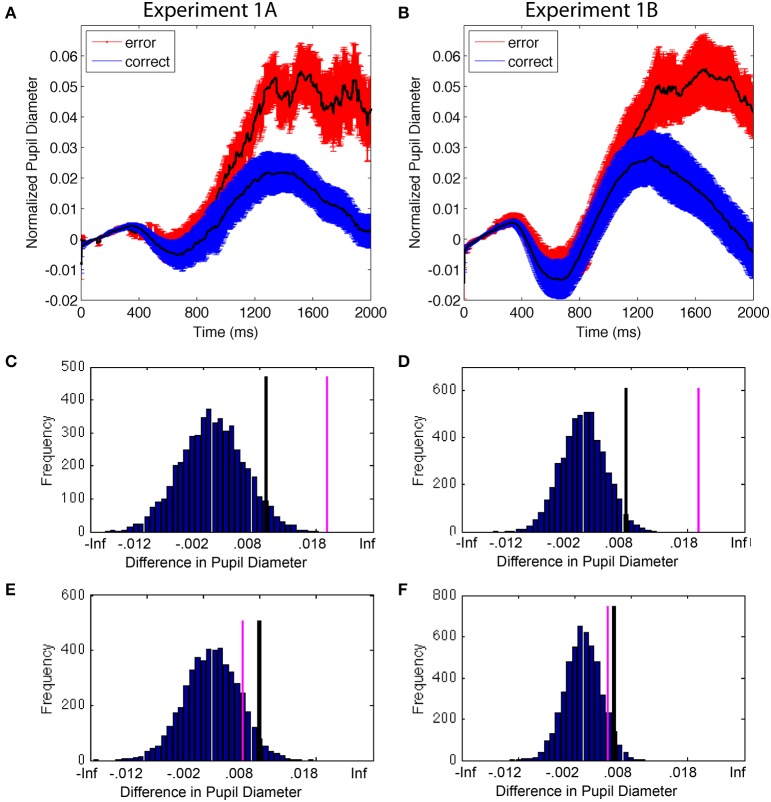
**(A,B)** Mean change in pupil diameter on correct and error trials. Error bars are standard error of the mean. **(C,D)** Permutation results on the difference between pupil diameter as a function of accuracy during the cognitive period; the difference had a <1% probability of occurring due to chance. Black lines identify the top 5% of the permutation results; pink lines are the actual data. **(E,F)** Permutation results during the control time period; the results had a >5% probability of occurring due to chance, suggesting that difference was not reliable.

Accuracy and RT are two ways to measure selection uncertainty due to internal variability. Trials in which subjects are less focused on the task produce longer RTs and a higher likelihood of an error (Weissman et al., 2006b; Smallwood and Schooler, 2015). First, we examined whether pupils would be larger overall on error trials compared to correct trials: although not all trials that involve uncertainty will result in errors, trials with the greatest uncertainty have a higher probability of resulting in errors. Therefore, we expected greater average pupil diameter on error trials than correct trials (Figure 4). Permutation analyses confirmed this pattern and showed that the difference in the stimulus-evoked pupil diameter between error and correct trials had <0.01% likelihood of occurring due to chance (Figures 4C,D). This was true for both experiments. In contrast, the difference between correct and error trials during the control time period was within the 95% distribution of permuted results (0.15 and 0.11 probability for E1A and E1B, respectively) (Figures 4E,F). The results replicate that of Smallwood et al. (2011) and Geva et al. (2013) and are consistent with the notion that pupils dilate when the subject experiences greater uncertainty in attentional selection. Importantly, this difference in pupil size was only present in the cognitive phase of the trial and not the control (sensory) time period, suggesting that the difference between correct and error trials was based on cognitive variability.

**Figure 5:**
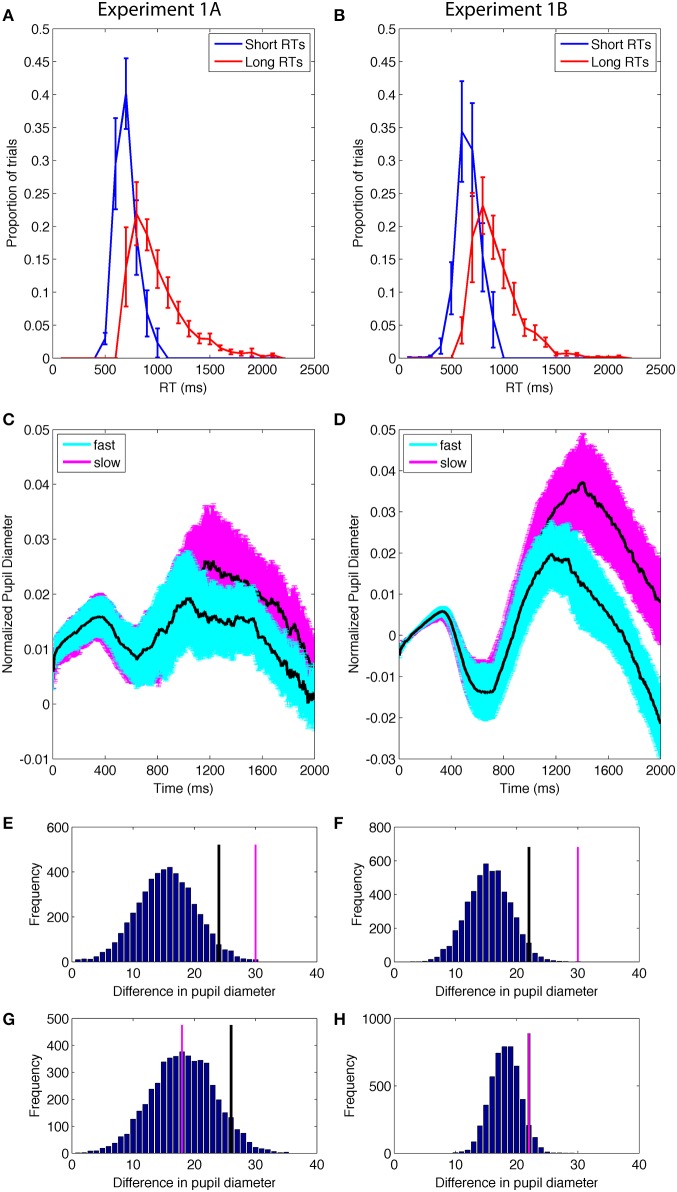
**(A,B)** Distribution of short and long RTs determined by a median split of data from correct trials within each individual. **(C,D)** Pupil diameter as a function of the RT median split. Error bands are standard error of the mean. **(E,F)** Permutation results on the difference between pupil diameter on trials with shorter and longer RTs during the cognitive period; the difference had a <1% probability of occurring due to chance. Black lines identify the top 5% of the permutation results; pink lines are the actual data. **(G,H)** Permutation results on the difference in pupil diameter as a function of RT from the control time period; the results had a >5% probability of occurring due to chance, suggesting that difference was not reliable.

Next, in order to test a more specific relationship between pupil diameter and uncertainty from internal fluctuations, we conducted a median split on individual RTs and compared the short and long RTs (Figures 5A,B). The difference in RT was significant {RT: E1A: *t*_(11)_ = 8.7, *p* < 0.0001, mean of difference [95% CI] = 294.3 [219.9, 368.7]; E1B: *t*_(12)_ = 11.7, *p* < 0.0001, mean difference [95% CI] = 257.7[209.9, 305.6]}.

The pupil data from the short RT and long RT trials were then compared using the permutation analysis approach described above. The average of the pupil data from the short RT and long RT trials were calculated for the two time periods (see Methods) (Figures 5C,D). Permutation analyses of the difference between short- and long-RT trials indicated that the likelihood of the difference arising from chance, given the current data, was < 0.01 in both E1A and E1B (Figures 5E,F). Long RT trials were accompanied by larger stimulus-evoked changes in pupil diameter than short RT trials and this difference was reliable in both experiments. In contrast, data from the control time period (1–1000 ms) from the same data had a >0.05 probability of occurring given the data (E1A = 0.56; E1B = 0.08), indicating that pupil diameter was not different between the long- and short-RT trials during the first 1000 ms after the visual search display appeared (Figures 5G,H).

In addition to comparing the average pupil diameter as a function of the RT median split, we also conducted an individual trial analysis in which the pupil data from all correct trials were rank-ordered by RT (shortest to longest) for each individual. We then conducted a correlation analysis on the average rank-ordered pupil diameter and RT. This resulted in a significant correlation in both experiments (Pearson's ρ E1A = 0.21, *p* < 0.001; E1B = 0.38, *p* < 0.0001). There was a significant linear relationship between pupil size and RT such that larger pupil diameters were associated with longer RTs, amongst only correct trials.

#### The effect of accuracy on the next trial

The previous data indicated that pupil diameters were larger when an error was made. However, those data beg the question of whether the change in pupil size reflected uncertainty in selection on the current trial, or if it was due to adjustments in cognitive performance based on outcomes in the previous trial (i.e., increasing vigilance or reinstating sustained attention in response to error monitoring). If the former, then we would expect no carryover effects from one trial to the next; if the latter, we would expect changes in pupil diameter on one trial to be accompanied by an improvement in performance on the next trial.

The difference in pupil diameter on error trials lasted well into the next trial, producing baseline differences; however, there was no difference in the stimulus-evoked pupil diameter on trials following correct vs. error trials. In behavior, accuracy of the previous trial (and the difference in the pre-stimulus baseline pupil diameter) was not accompanied by better performance on the next trial: RT was slower, but accuracy was lower (E1A) or statistically equivalent (E1B) following an error trial, RT: E1A: *t*_(11)_ = 3.1, *p* < 0.01, mean diff[95% CI] = 37.0[108, 63.1]; E1B: *t*_(12)_ = 2.8, *p* < 0.05, mean diff[95% CI] = 58.8[12.3, 105.2]; Accuracy: E1A: *t*_(11)_ = 2.8, *p* < 0.05, mean diff [95% CI] = 0.05[0.006, 0.06]; E1B: *t*_(12)_ = 1.7, *p* = 0.11, mean diff[95% CI] = 0.05[−0.1, 0.10]. The presence of post-error slowing without a concomitant increase in performance in this study may have been due to the specific experimental parameters used, including relatively long ISIs (Danielmeier and Ullsperger, 2011). Results from both experiments demonstrate that while there was a long-lasting effect of the difference in pupil dilation that was accompanied by longer RTs on the next trial, there was no related performance enhancement on the next trial. This suggests that the dilation in pupil diameter on error trials likely reflected processing of the current stimulus, which carried over into the next trial, rather than changes in cognitive control in preparation of behavioral adjustments on the next trial.

#### The effect of accuracy on pupil time-locked to the response

**Figure 6:**
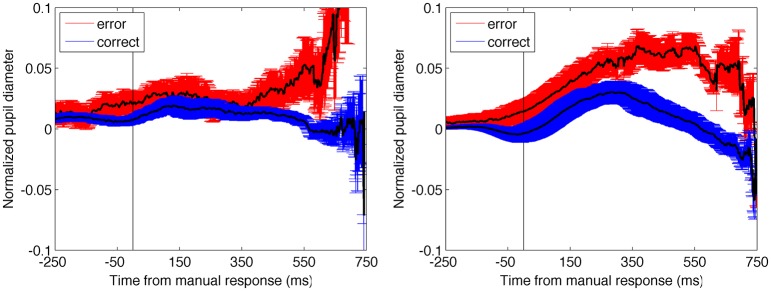
**Pupil diameter time-locked to the manual response as a function of trial accuracy**. Error bars are standard error of the mean. In both experiments, the results from the permutation analysis were the same for the pre-response and post-response time periods, suggesting that changes in the pupil data were not time-locked to the manual response. Compare with stimulus-locked data plotted in Figure 4.

We next conducted an analysis to understand whether the change in pupil diameter could be better understood as being more related to the response than ongoing selection. Data from 500 ms prior to the response up to 1000 ms after the response were analyzed in a similar manner to the stimulus-evoked accuracy data, which involved the clearest binary division of data (Figure 6). The permutation analysis resulted in a non-significant difference between both the post-response and pre-response periods in E1A (9 and 16% probability of occurrence, respectively) and significant differences in both time periods in E1B (< 0.01% chance in both cases). While the data from the two experiments diverge in this analysis, the finding that the pre-response and post-response periods produced the same result within each experiment suggests that the change in pupil diameter associated with accuracy was not time-locked to the manual response. The change in pupil data, therefore, appears to be better explained by timing associated with the stimulus-onset.

#### Summary of results

Together, the data from E1AB suggest that changes in pupil diameter were sensitive to the degree of selection uncertainty in response to the visual search task. Moreover, this uncertainty was not specific to any particular experimental condition, but rather occurred across all conditions as a function of RT and accuracy. Differences in pupil diameter were most pronounced between error and correct trials, but pupil diameter also scaled with RT on correct trials, suggesting that pupils were highly sensitive to increased mental effort related to performance monitoring and the efficiency of attentional selection.

## Experiment 2

In the previous experiments, changes in pupil diameter were larger on trials with longer RTs and when errors were made. We hypothesized that the change in pupil diameter reflected uncertainty in the attentional selection of the target on the current trial. We sought to find additional evidence that the change in pupil diameter was related to uncertainty regarding target selection. To do so, we increased the difficulty of the search task in Experiment 2 by adding distractors and a pattern mask. Additionally, we manipulated the presence or absence of feedback on alternating blocks in order to compare directly the effect of feedback, which was a between-subjects manipulation in E1AB. Thus, Experiment 2 increased selection uncertainty overall by increasing difficulty while maintaining the presence (or absence) of feedback. Search difficulty was expected to increase selection uncertainty overall, but we hypothesized that the effect on pupil diameter would be most evident on correct trials. This was based on the reasoning that errors in both experiments would involve similar levels of uncertainty (e.g., due to mind-wandering), but that differences in uncertainty on correct trials would be primarily due to task difficulty.

### Methods

#### Participants

Nineteen new subjects from the University of California, Davis participated in this experiment (12 females; 18–25 years). Data from five subjects were excluded due to chance performance, leaving 14 subjects in the analysis. Each subject received course credit for participating. The experimental protocol was approved by the Internal Review Board at UC Davis.

#### Apparatus and stimuli

The apparatus and stimuli were the same as in Experiment 1, with the following exceptions: The search display had four stimuli oriented in an equidistant circular formation around the fixation cross (Figure 1). The luminance of all stimuli was always identical and equivalent to the low contrast stimuli in Experiment 1. The location of the target in each of the possible four locations was random. The search display was followed by a pattern mask that terminated sensory processing.

#### Procedure

The conditions of the testing environment as well as the setup were identical to the previous experiment. Trials began as in E1AB with the fixation cross in the center of the screen, followed by presentation of the stimuli for a predetermined amount of time based on individual performance in a staircase procedure. Upon completion of the staircase, the experimenter chose a presentation time such that accuracy would be approximately 70%. Subjects had break periods between each block of trials.

On each experimental trial, the search stimuli were replaced with a pattern mask until the subject responded, at which point the next trial began. The experiment consisted of six blocks each with 60 trials. Subjects received auditory feedback on alternate blocks, beginning with a no-feedback block. Feedback was the only within-subject manipulation in this study. On feedback blocks, a high tone (800 Hz) was played after correct responses, and a low tone (200 Hz) after incorrect responses. These were identical to those from E1B.

### Results

#### Effect of feedback as a within-subject manipulation

RT data from feedback and no-feedback blocks were compared using a paired *t*-test. RTs were significantly shorter when feedback was present, *t*_(13)_ = 4.3, *p* < 0.001, mean diff [95% CI] = 107.6 [53.4, 161.7]. The difference between accuracy was also significant, *t*_(13)_ = 2.6, *p* < 0.05, mean diff[95% CI] = 0.03 [0.0055, 0.05]. This indicated that the presence of feedback made responses faster and more accurate.

Permutation analyses on trials with and without feedback indicated that stimulus-evoked pupil diameter was larger on feedback trials both during the control period as well as the cognitive time periods. This was true even though the data were normalized such that the baseline differences between conditions were accounted for. Consistent with this, the pre-stimulus baseline pupil diameter was also larger on feedback blocks, *t*_(13)_ = 3.2, *p* < 0.01; mean diff [95% CI] = 0.05 [0.01, 0.07], suggesting that feedback enhanced behavioral performance by increasing arousal and attentiveness and that this effect produced larger pupil diameters over the entire block (Aston-Jones and Cohen, 2005; Gilzenrat et al., 2010; Smallwood et al., 2011).

#### Comparison between experiments

The primary motivation for this experiment was to examine the effect of increased difficulty on the stimulus-evoked change in pupil diameter. Consistent with expectations that increasing the number of distractors would increase search difficulty, accuracy was significantly lower in E2 than in E1AB, E1A = 0.82, E1B = 0.82, E2 = 0.72; *F*_(2, 36)_ = 4.1, *p* < 0.05, η^2^ = 0.19, *post-hoc t*-tests comparing accuracy from E2 to E1A and E1B: *t*_(24)_ = 2.8, *p* < 0.01, mean diff[95% CI] = 0.10 [0.03, 0.18]; *t*_(25)_ = 2.5, *p* < 0.05, mean diff[95% CI] = 0.10 [0.02, 0.19], *t*-test comparing E1A and E1B, *t*_(23)_ = 0.02, *p* = 0.98. In addition to overall accuracy being lower, we hypothesized that RTs would be longer with increased difficulty, particularly on correct trials, because the decision boundary would take longer to reach. We did not expect the same difference to be present on error trials because there may be many reasons for an error that are unrelated to the decision process. Consistent with this, RTs for *correct* trials were significantly shorter in E1A and E1B compared to E2 {both *t*_(24)_ = 3.2, *p* < 0.01; smallest mean diff[95% CI] = 183.8 [64.9, 302.6]}; there was no difference between E1A and E1B [*t*_(23)_ = 1.1, *p* = 0.26]. When the response was correct, the decision was arrived at sooner when the task was easier, irrespective of feedback. However, the difference in RT on error trials between experiments was not significant (all *t* < 1.8, *p* > 0.05), suggesting that the RT difference on correct trials was not due to E2 subjects having shorter RTs overall. The behavioral results demonstrate that E2 was more difficult overall and that the greatest difference in performance uncertainty occurred on correct trials.

We next examined the pupil data. We hypothesized that the harder search task in E2 would elicit greater uncertainty in target selection than the easier search tasks in E1AB on correct trials overall, but that this difference in uncertainty would be most apparent on correct trials. This is because correct trials mostly reflect on-task behaviors that are affected by experimentally-induced uncertainty. On the other hand, error trials may occur for a number of task-unrelated reasons that affect uncertainty (e.g., mind-wandering). We therefore hypothesized that pupil diameters should be larger in E2 on correct trials, but similar between experiments on error trials. To test this hypothesis, we compared the difference data between error and correct trials for data with no-feedback (i.e., E1A vs. E2_nofeedback_) and data with feedback (i.e., E1B vs. E2_feedback_). Permutation analysis confirmed that the difference was significantly larger in E1A and E1B compared to the equivalent feedback conditions in E2 (i.e., E2_nofeedback_, E2_feedback_). The probability of the difference being due to chance was <0.5%.

**Figure 7:**
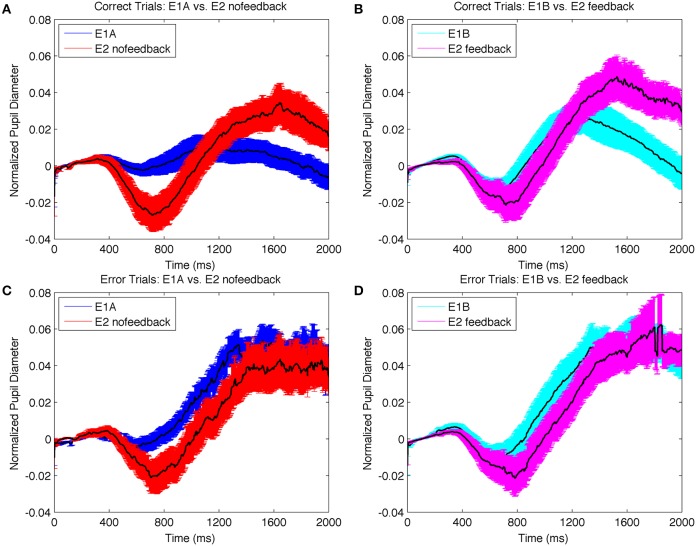
**Change in normalized pupil diameter on correct trials plotted together for: (A) E1A and E2_nofeedback_ (B) E1B and E2_feedback_; and on error trials for (C) E1A and E2_nofeedback_ (D) E1B and E2_feedback_**. The difference between experiments were more pronounced on correct trials compared to error trials, suggesting that increased attentional uncertainty in the more difficult search task produced larger task-related changes in pupil size.

We next evaluated the more specific hypothesis that pupil diameter should be greater on correct trials in E2 compared to E1AB, but should be similar on error trials. To do so, we conducted separate permutation tests on the correct and error trials from the no-feedback and feedback experiments separately (Figure 7). Consistent with expectations, pupil diameters were larger on correct trials in E2 compared to E1; the permutation analysis indicated that the size of the difference had only a 4.4 and 7.5% probability of being due to chance for no-feedback and feedback conditions respectively; in contrast, the likelihood of the same difference being due to chance on error trials was 81.5 and 64.5%. Together, these results suggest that the more difficult visual search task in E2 produced greater selection uncertainty, resulting in greater differences in pupil diameters on correct trials, when differences between experimental task demands were most evident. Thus, the difference in pupil diameter based on accuracy appears to be less about being correct vs. incorrect, *per se*, but rather reflects the amount of uncertainty in target selection on those trials.

### Discussion

In these experiments, we measured changes in pupil diameter as a function of accuracy, feedback, and difficulty during visual search. The purpose of the experiments was to understand the source of changes in pupil diameter during visual search. Contrary to expectations, we did not see systematic changes in pupil diameter due to our experimental conditions based on target and distractor saliency. However, we found systematic increases in the stimulus-evoked pupil diameter when subjects appeared to experience greater uncertainty in target selection, as indexed by longer RTs and errors. These results led us to conclude that increases in pupil diameter did not reflect a specific stimulus condition, but was rather related to the uncertainty involved in selecting and discriminating the target on a given trial. These data are compatible with earlier accounts of changes in pupil size being due to increased mental effort and response uncertainty (Kahneman and Beatty, 1966; Beatty, 1982; Richer and Beatty, 1987); here we extend those findings to visual search situations in which uncertainty is measured continuously with RT and culminating in response accuracy. We suggest that uncertainty is the source of the increase in mental effort and that pupil size reflects this relationship.

In E1AB, we found that task-evoked pupils were larger on correct trials with longer RTs and also larger overall on error trials compared to correct trials. We hypothesized that larger stimulus-evoked pupils correlated with RT on correct trials because of internal fluctuations in attentiveness that affected the degree of uncertainty in target selection when the search display appeared. Internal sources of uncertainty could occur for a host of reasons, including mind-wandering and reductions in vigilance (Gabay et al., 2011; Smallwood et al., 2011). On more extreme trials, such decreases in task vigilance will have a high likelihood of resulting in an error, but fluctuations in preparatory attentiveness even on correct trials should covary with RT.

In E2, we increased difficulty and manipulated the presence of feedback from block-to-block. We found that feedback produced larger pupil sizes overall (perhaps signaling greater arousal), but that the difference in pupil diameter between correct and incorrect trials was present in both conditions. Thus, the change in pupil due to feedback was independent of the changes related to accuracy. More importantly, we were interested in comparing pupil diameter in E2 with E1AB in order to test the effect of selection difficulty on the difference in stimulus-evoked pupil on correct and error trials. We hypothesized that a more difficult task would produce greater uncertainty, particularly on correct trials, and therefore we should find a smaller difference between correct and error trials in E2 compared to E1AB. The interaction between experiment and pupil diameter on correct and error trials was indeed significant, suggesting that greater task difficulty increased selection uncertainty on all trials, resulting in more similar pupils on correct and error trials. Moreover, this effect was due primarily to larger pupils on correct trials in E2, rather than smaller pupils on error trials.

We conclude from our data that it is selection uncertainty that triggers an increase in mental effort and cognitive control mechanisms associated with increased pupil diameter. This is based on the reasoning that if the increase in pupil reflected mental effort itself, then we would expect it to be associated with better performance; however, we find that larger pupils were related to decreases in performance (longer RTs and errors). It remains possible, however, that the change in pupil diameter reflects reactive mental effort that was initiated by uncertainty, rather than being a direct consequence of uncertainty itself. The current data are compatible with both models. Additionally, our characterization of selection uncertainty is also similar to earlier descriptions of response uncertainty (Richer and Beatty, 1987). However, our data cannot be entirely explained by response uncertainty, given that pupils were larger when more distractors were present (E2 compared to E1) even though both tasks involved the same two alternative forced choice (2AFC) response. This suggests that pupil diameter may be a sensitive measure of uncertainty in a variety of cognitive domains.

Uncertainty and mental effort may be inextricably linked with pupil diameter if uncertainty triggers phasic activity in the LC-NE system associated with prefrontal cognitive control mechanisms that may help improve the likelihood of correct performance (Minzenberg and Carter, 2008). The necessity of prefrontal cognitive control mechanisms is conceptually equivalent to earlier characterizations of the need to increase “mental effort” commensurate with cognitive processing load (Hess and Polt, 1964; Kahneman and Beatty, 1966; Kahneman, 1973; Beatty, 1982). In this study, we hypothesize that mental effort was expressed by the need to reinstate cognitive control mechanisms that maintain goal-relevant information and actions (i.e., to find a particular target) on trials where uncertainty was high. These cognitive control functions are known to be highly dynamic and flexible, rapidly adjusting behaviors to external stimuli in response to internal goals (Miller and Cohen, 2001; Braver et al., 2009). However, our data do not speak directly to the validity of the LC-NE model and therefore it is necessary to conduct new studies to flesh out the relationship between uncertainty, mental effort, and cognitive control in the brain.

The pupil data in the current studies demonstrated performance-related changes in stimulus-evoked pupil that can be used as an implicit measurement of the uncertainty associated with attentional selection. The implicit nature of the pupil signal has an advantage over methods that rely on overt measurements such as RT or accuracy. Importantly, uncertainty in attentional selection is a continuous dimension that can be influenced by a number of internal (e.g., mind-wandering, lapses in vigilance) and external (e.g., decisional difficulty, perceptual similarity) factors that will increase the time to make a response, with the most extreme cases resulting in an error. These data suggest that the stimulus-evoked pupil diameter can be taken as a continuous metric of uncertainty that correlates with accurate RTs and errors.

It is interesting to note that our hypothesis regarding changes in baseline pupil size as a function of the statistical structure of target and distractor saliency was not confirmed. This could be due to several reasons. First, it may be that larger fatigue effects masked smaller changes over time due to learning of the task structure. Alternatively, it may be that our task did not sufficiently invoke learning mechanisms since trial-by-trial performance was not directly tied to learning the statistics of target and distractor saliency. Finally, it may be that the bottom-up saliency effects on attention did not increase selection uncertainty in this task. In contrast, the results demonstrated clear changes in task-evoked pupil diameter that can be used as an index of uncertainty in attentional selection during visual search that were independent of the exact stimulus conditions.

### Conflict of interest statement

The authors declare that the research was conducted in the absence of any commercial or financial relationships that could be construed as a potential conflict of interest.
